# Utility of NT-proBNP for Identifying LV Failure in Patients with Acute Exacerbation of Chronic Bronchitis

**DOI:** 10.1371/journal.pone.0052553

**Published:** 2013-01-14

**Authors:** Qing-ping Wang, Xiao-zhi Cao, Xue-dong Wang, Juan Gu, Li-min Wen, Li-ming Mao, Ping-nan Shan, Ai-guo Tang

**Affiliations:** 1 Department of Clinical Laboratory, The Fifth People's Hospital of Wuxi, Affiliated Hospital of Nanjing Medical University, Wuxi, Jiangsu, PR China; 2 Department of Clinical Laboratory, The Shaoxing Hospital of China Medical University and Central Hospital of Shaoxing County, Shaoxing County, Zhejiang, PR China; 3 Department of Caroliology, Fuzhou General Hospital, Nanjing Command, PLA, Fuzhou, Fujian, PR China; 4 Department of Clinical Laboratory, The Second XiangYa Hospital of Central South University, Changsha, Hunan, PR China; Gentofte University Hospital, Denmark

## Abstract

**Background:**

NT-proBNP has been widely regarded as a useful tool for diagnosis or exclusion of heart failure (HF) in many settings. However, in patients with acute exacerbation of chronic bronchitis (AECB), its roles have not been well described. The objective of this study was to evaluate the diagnostic performance of NT-proBNP for identifying left ventricular (LV) failure in such patients.

**Methods and Results:**

311 AECB patients and 102 stable chronic bronchitis patients with no history of HF were enrolled. Plasma NT-proBNP concentrations were measured using Roche Elecsys. The European Society of Cardiology (ESC) diagnostic principles were adopted to identify HF and the diagnostic performance of NT-proBNP was evaluated by ROC. Our results showed, the median NT-proBNP level in patients with LV failure [4828.4 (2044.4–9203.6) ng/L] was significantly higher than that in those without LV failure [519.2 (179.1–1409.8) ng/L, *p*<0.001] and stable controls [207.5 (186.5–318.2) ng/L, *p*<0.001]. LV failure, renal function, atrial fibrillation and systolic pulmonary artery pressure were independent predictors of NT-proBNP levels (all *p*<0.05). The area under ROC curve (AUC) of NT-proBNP for identifying LV failure was 0.884, significantly superior to clinical judgment alone (AUC 0.835, *p* = 0.0294). At the optimal cutoff value of 935.0 ng/L, NT-proBNP yielded sensitivity 94.4%, specificity 68.2%, accuracy 74.3% and negative predictive value 97.6%. Adding the results of NT-proBNP to those of clinical judgment improved the diagnostic accuracy for LV failure.

**Conclusion:**

As a tool for diagnosis or exclusion of HF, NT-proBNP can help physicians identify LV failure in patients with AECB.

## Introduction

Acute exacerbations of chronic bronchitis (AECB) are episodes of difficulty in breathing in a patient with chronic bronchitis (CB). They are common causes of morbidity and in patients with concomitant airway obstruction (COPD) are major causes of mortality [1]–[4]. Although infectious agents (bacterial or viral infection) are estimated to account for around 50–70% of these episodes, non-infectious agents may cause worsening of CB [5]. Of these non-infectious etiologies, heart failure (HF) is a potential trigger for AECB [6]–[8]. Yet, a diagnosis of HF in such patients is challenging, because the symptoms and signs of HF may overlap with those of CB [7], [8]. Moreover, in many of these situations, physical exam findings, lab tests, chest X-rays and cardiac function tests (in particular, echocardiography) are often nonspecific or not always available [2], [7], [8]. As a result, missed diagnosis of HF is frequent.

N-terminal pro–brain natriuretic peptide (NT-proBNP) is a biologically inactive fragment of B type natriuretic peptide (BNP). Previous studies have reported that NT-proBNP may be regarded as a useful tool for diagnosis or exclusion of HF [9]–[12], and shown to perform well in distinguishing acute dyspnea between cardiac origin and pulmonary causes in many settings [13]–[17]. However, the production, secretion and probably even the peripheral metabolism of B type natriuretic peptides are closely regulated by several neuro-hormones and immunological factors [18], as well as by a number of other factors such as renal function and clinical conditions [13], [19], [20]. Hence, it is unclear whether they retain their diagnostic utility for HF in patients with AECB. In a single-center retrospective cohort study, *Gariani et al.* [21] assessed the diagnostic performance of BNP for detecting LV dysfunction in patients with acute exacerbation of COPD and concluded that BNP could help physicians in identifying HF. However, NT-proBNP is different in biological activity from BNP, and has a longer half-life, with plasma levels 2–10 times higher than BNP [22]. Another study suggested NT-proBNP was useful in acute exacerbation of COPD associated with left ventricular (LV) dysfunction, but they focused on the selected patients with such severe acute exacerbation of COPD as to require non-invasive or conventional ventilation [23]. Therefore, it is unclear what the diagnostic value of NT-proBNP would be in cases of mild presentations of acute exacerbation of COPD. In addition, although CB is generally considered one of the two forms of COPD and reported in approximately 85% of patients with severe COPD, the syndrome of CB commonly occurs without airway obstruction [2]. Conversely, only a minority of patients with CB have COPD [2]. Thus, extrapolation of these results to NT-proBNP in patients with AECB should not be straightforward.

The current study was undertaken to assess the diagnostic performance, including sensitivity, specificity, accuracy, positive and negative predictive value of NT-proBNP for diagnosis of LV failure in patients with AECB.

## Materials and Methods

### Study populations

In this cross-sectional study, all consecutive patients presenting to the Emergency Department (ED) with a primary diagnosis of AECB were screened. An AECB is a clinical diagnosis made when a patient with chronic bronchitis experiences a sustained (e.g., 24–48 h) increase in cough, sputum production, and/or dyspnea. Patients with a known history of HF (ascertained by patient self-report and review of the prior medical record at the time of the ED visit), acute myocardial infarction, or overt causes of dyspnea (including chest wall trauma or penetrating lung injury), or because echocardiography was not feasible were excluded. In addition, patients hospitalized in a cadre ward with a stable phase of chronic bronchitis but unlikely to be associated with HF (no history of, or risk factors for, LV disease, a normal pertaining to HF in the physical examination or on chest radiograph; and a normal echocardiograph) were recruited as controls. This study was performed from August 2010 to December 2011, the institutional review board of the Shaoxing Hospital of China Medical University approved the protocol and written, informed consent was obtained from all patients.

### Study protocols

Thorough clinical examinations, including medical history, physical examination, chest X-ray, ECG, echocardiography, pulmonary function tests were performed on every included patient. Echocardiographic examination was performed on the first day of admission and the following parameters were recorded: left ventricular ejection fraction (LVEF), systolic pulmonary pressure (SPAP), early diastolic inflow (E) and late atrial inflow (A) velocities, deceleration time (DT), and LV isovolumetric relaxation time (IVRT). Pulmonary function tests, including forced vital capacity (FVC), forced expired volume in one second (FEV1) and FEV1/FVC ratio, were performed during a remission stage. The final interpretation of these outcomes was performed by radiologists and cardiologists who were blinded to NT-proBNP results at all sites.

Blood samples were taken within 24 h of admission to analyze plasma NT-proBNP, serum creatinine, and arterial blood gas. Renal function was assessed by using MDRD equation to estimate glomerular filtration rate (eGFR). eGFR(mL/min/1.73 m^2^) = 175×[serum creatinine (µmol/L)×0.0113]^−1:154^×age^−0.203^×0.742 (if patient is female) [24].

Using all available information except NT-proBNP levels and echocardiographic data, the ED physicians estimated the probability of LV failure on a 0–100% visual analogue scale (VAS) as the clinical judgment for diagnosis of LV failure, with 0–20% indicated low probability of LV failure, 80–100% indicated the high probability of LV failure and VAS score of 21–79% indicated moderate probability of LV failure. This estimate was recorded for future comparison with NT-proBNP results.

### Final adjudicated diagnosis of LV failure

An expert panel (two cardiologists and one pulmonologists) that was blinded to the results of the measurements of NT-proBNP made the final adjudicated diagnosis. In case of no consensus, the majority decided whether the case definition was met and the final diagnosis was confirmed at discharge. To diagnose LV failure, the European Society of Cardiology (ESC) diagnostic principles (i.e., subjective symptoms supported by echocardiographic evidence of LV (systolic and/or diastolic) dysfunction) were adopted [25]. LV systolic failure was defined as the presence of symptoms in combination with an LVEF<45%. For LV diastolic failure, patients had to have indicative symptoms and signs of HF in combination with following echocardiographic LV diastolic dysfunctions: (1) Impaired relaxation: E/A ratio<0.8, DT>200 ms and IVRT>100 ms, a change in E/A ratio with the Valsalva maneuver<0.5; (2) Pseudonormal: E/A = 0.8–1.5, DT = 160–200 ms, IVRT = 60–100 ms, a change in E/A ratio with the Valsalva maneuver≥0.5; (3) Restrictive filling: E/A≥1.5, DT<160 ms and IVRT<60 ms, a change in E/A ratio with the Valsalva maneuver≥0.5.

### NT-proBNP test

Blood samples were collected by venipuncture into ethylene diamine tetra acetic acid (EDTA) tubes. The samples were centrifuged and plasmas were then analyzed within 2 h using a monoclonal electro-chemiluminescence immunoassay, performed on a Roche Elecsys 2010 automated platform (Roche Diagnostics, Basel, Switzerland). The assay has an effective measuring range of 5–35 000 ng/L, the within-run coefficient of variation (CV) was 2.7% at a concentration of 175 ng/L and 1.9% at 1068 ng/L, the between-run CV were 3.4%, 5.4%, and 4.3% at levels of 37.8, 236.3, and 473.2, respectively.

### Statistical analysis

Statistical analysis of all data was performed using SPSS 13.0 (SPSS Inc., Chicago, IL) program. Continuous data were presented as medians with interquartile range (IQR). Categorical data were presented as a proportion (%). Median comparisons were made using *Mann-Whitney U* test. Chi square or Fisher's exact test was used to compare the proportions between groups. The relationship between NT-proBNP levels and continuous variables such as age and eGFR were examined by rank correlation analysis, whereas categorical variables such as gender and atrial fibrillation were assessed by *Mann-Whitney U* tests.

Multivariate linear regression analysis was performed to identify the significant independent predictors of NT-proBNP levels, and multivariate logistic regression analysis was used to identify the association of demographic and clinical variables with HF. For these multivariable analyses, variables were selected stepwise to reduce the model to only statistically significant parameters. That is, enter significant variables sequentially; after entering a variable in the model, check and possibly remove variables that became non-significant (*P*>0.1); when none of the remaining variables were significant at the 0.05 level, selection was terminated.

The diagnostic performance of plasma NT-proBNP identifying LV failure from AECB patients was evaluated by using a receiver operating characteristic curve (ROC), the overall discriminatory ability of NT-proBNP was shown by the area under the curve (AUC). We also calculated sensitivity, specificity, accuracy, positive and negative predictive values. *P*<0.05 was considered statistically significant.

## Results

In this study, 311 AECB and 102 stable CB patients without history of HF were finally included. There were no significant differences in these demographic and clinical characteristics between the patients with AECB and those with stable CB, except for the arterial partial pressure of carbon dioxide (PaCO_2_) and oxygen (PaO_2_). The demographic and clinical characteristics of the 311 AECB and 102 stable CB patients are shown in Table 1. In patients with AECB, the overall rate of LV failure was 23.2%. Compared to patients without LV failure, those with LV failure had a more frequent abnormality on ECG, atrial fibrillation, coronary heart disease and had a significantly higher PaCO_2_, lower PaO_2_ and LVEF. No significant differences were observed on other demographic and clinical characteristics between the both subgroups.

**Table 1:** Demographic and clinical characteristics of patients with AECB or stable CB.

Variables	AECB			Stable CB (n = 102)
	LV failure (n = 72)	Non-LV failure (n = 239)	All (n = 311)	
Age (year)	77.0 (67.0–81.5)	75.0 (69.0–80.0)	75.0 (68.0–80.0)	74.5 (69.0–81.0)
Male gender	73.6%	73.2%	73.3%	73.5%
eGFR (mL/min/1.73 m2)	69.0 (56.4–88.4)	73.4 (59.2–89.0)	72.8 (58.6–89.0)	75.2 (60.0–89.7)
pH	7.39 (7.34–7.43)	7.40 (7.37–7.43)	7.40 (7.37–7.43)	7.40 (73.5–7.44)
PaO_2_(mm Hg)	68.0 (54.5–78.0)	72.0 (63.0–84.8)Δ	71.0 (61.3–83.0)	82.5 (78.0–90.5)⋇
PaCO_2_(mm Hg)	54.5 (40.5–69.5)	45.0 (41.0–54.8)Δ	45.0 (41.0–58.0)	40.7 (37.5–43.2)⋇
LVEF (%)	41.7 (31.7–51.7)	60.0 (56.5–63.5)Δ	59.0 (52.0–64.0)	60.6 (58.0–64.0)
FEV1/FVC	73.0 (67.0–79.0)	74.0 (68.5–79.5)	74.0 (68.0–80.5)	75.5 (66.5–80.5)
SPAP (mm Hg)	43.5 (34.0–55.0)	41.0 (31.0–52.0)	44.0 (32.5–50.5)	40.0 (30.0–52.5)
Abnormality on ECG*	59.7%	30.1%Δ	37.0%	31.4%
LV systolic failure	69.4%		16.1%	
LV diastolic failure	30.6%		7.1%	
Atrial fibrillation	16.7%	6.3%Δ	8.7%	4.9%
Coronary heart disease	13.9%	4.6%Δ	6.8%	2.9%
Cor pulmonale	34.7%	31.0%	31.8%	32.4%
Diabetes mellitus	12.5%	10.9%	11.3%	12.7%
Hypertension	44.4%	40.6%	41.5%	39.2%
Bronchiectasis	10.0%	9.3%	9.3%	10.8%

Data are expressed as median (IQR), unless otherwise specified.

AECB: acute exacerbation of chronic bronchitis; CB: chronic bronchitis; LV: left ventricular; eGFR: estimated glomerular filtration rate; PaO_2_: artery partial pressure of oxygen; PaCO_2_: artery partial pressure of carbon dioxide; LVEF: Left ventricular ejection fraction; FEV1: forced expired volume in one second; FVC: forced vital capacity; SPAP: systolic pulmonary artery pressure.

* Presence of Q wave, ST depression, ST elevation or T wave inversed.

Δ *p*<0.05, compared to patients with LV failure;

⋇ *p*<0.05, compared to all patients with AECB.

### 1. NT-proBNP levels in study population

**Figure 1:**
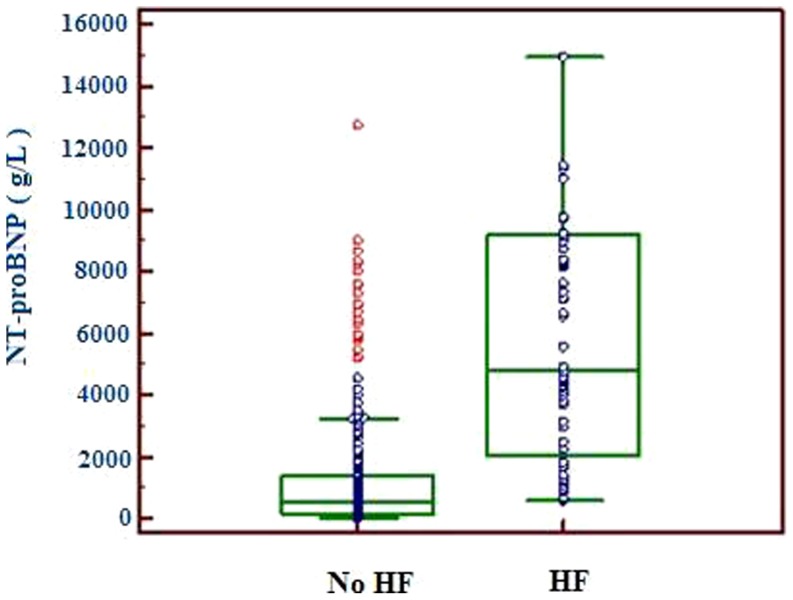
Box plot of plasma NT-proBNP concentrations in AECB patients with or without LV failure. Horizontal lines in the box indicate 25th, 50th and 75th percentiles, and I bars represent highest and lowest values. NT-proBNP: N-terminal pro-B type natriuretic peptide; AECB: acute exacerbation of chronic bronchitis; LV: left ventricular.

The median concentration of NT-proBNP in all AECB patients was 839.6 ng/L (247.6–3334.0 ng/L), significantly higher than that in those with stable CB [207.5 (186.5–318.2) ng/L, *p*<0.001]. More than one half of the patients having NT-proBNP values above current age recommended cutoffs (125 ng/L for patients aged≤75 and 450 ng/L for those aged>75), regardless of LV failure. Patients with LV failure had significantly higher median NT-proBNP concentrations [4828.4 (2044.4–9203.6) ng/L) compared to those without LV failure [519.2 (179.1–1409.8 ng/L, p<0.001) for those without LV failure and controls [207.5 (186.5–318.2) ng/L, *p*<0.001]. The distributions of NT-proBNP values in patients with and without LV failure are shown in Fig. 1.

### 2. Variables associated with plasma NT-proBNP

In order to assess whether variables other than LV failure were associated with plasma NT-proBNP in patients with AECB, we explored the relationship between NT-proBNP levels and other demographic and clinical variables using univariate analysis and the independent predictors of plasma NT-proBNP using multivariate linear regression analysis. Table 2 presents the variables significantly associated with NT-proBNP levels in both univariate analysis and multivariate linear regression analysis.

**Table 2:** Variables significantly associated with NT-proBNP in both univariate analysis and multiple linear regression analysis.

variable	Univariate *P* Value	Multiple regression	
		β-Coefficient	*P* Value
eGFR (mL/min/1.73 m2)	0.000	−0.289	0.000
Atrial fibrillation (yes or no)	0.001	0.207	0.005
SPAP (mm Hg)	0.001	0.108	0.016
HF (yes or no)	0.001	0.530	0.000

NT-pro BNP: N-terminal pro-B type natriuretic peptide; eGFR: estimated glomerular filtration rate; SPAP: systolic pulmonary artery pressure; HF: heart failure.

In univariate analysis, age (*r* = 0.227, *p*<0.001), eGFR (*r* = −0.274, p<0.001), LVEF (*r* = −0.35, *p*<0.001), systolic pulmonary artery pressure (*r* = 0.283, *p*<0.001), artery blood pH (*r* = −0.132, *p* = 0.020) and LV failure (*z* = 9.972, *p*<0.000), atrial fibrillation (z = 4.234, *p*<0.001), coronary heart disease (z = 2.979, *p* = 0.003), cor pulmonale (z = 5.417, *p*<0.001) were significantly correlated with NT-proBNP levels. In multivariate linear regression analysis, however, only LV failure, eGFR, atrial fibrillation and systolic pulmonary artery pressure were significant and independent predictors of plasma NT-proBNP levels (all *p*<0.05).

### 3. Ability for NT-proBNP to diagnose LV failure

**Figure 2:**
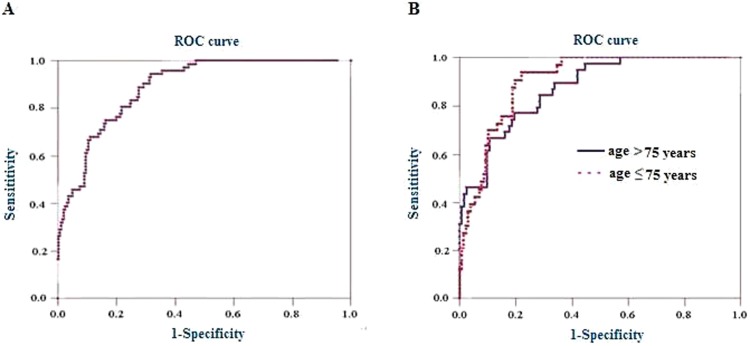
Receiver operating characteristic curve of NT-proBNP for diagnosis of LV failure in patients with AECB. (A) All 311 patients included in the present study. The area under the receiver operating characteristic curve is 0.884 (95% confidence interval, 0.847 to 0.920) for all patients; (B) Patients stratified by age≤75 or>75 year. The AUC is 0.902 (95% confidence interval 0.846 to 0.944) for patients aged≤75 year, 0.872 (95% confidence interval 0.771 to 0.894) for patients aged>75 year.

Using the final diagnosis of HF as a “gold standard”, we constructed a ROC plot for NT-proBNP as a diagnostic test in identifying LV failure (Fig. 2A) and determined the optimal diagnostic cutoff values based on a weighted Youden index. Results demonstrated that, the area under the ROC curve (AUC) in all patients was 0.884 (95% confidence interval 0.847 to 0.920, *p*<0.0001), the optimal cutoff value for NT-proBNP to diagnosis LV failure was 935.0 ng/L. At this cutoff value, NT-proBNP had a sensitivity 94.4%, specificity 68.2%, accuracy 74.3% and resulted in a negative predictive value of 97.6%. At a cutoff value of 584 ng/L, NT-proBNP could rule out 126/311 (40.5%) of patients with a negative predictive value of 100%.

We further obtained ROC curves according to the current recommended age stratification (aged≤75 and aged>75) [26] (Fig. 2B). The AUCs for patients aged≤75 and those aged>75 were 0.902 (95% confidence interval 0.846 to 0.944, *p*<0.0001) and 0.872 (95% confidence interval 0.771 to 0.894, *p* = 0.0001) respectively. At the optimal cutoff value 947.0 ng/L for patients aged≤75, NT-proBNP had sensitivity 93.9%, specificity 78.0%, accuracy 81.3% and negative predictive value 98.0%. At the optimal cutoff value 2241.0 ng/L for those aged>75, the sensitivity, specificity, accuracy and negative predictive value were 76.9%, 80.4%, 79.5 and 90.9% respectively. The accuracies of NT-proBNP at their corresponding optimal cutoff value for LV failure in patients stratified by age were slightly higher than that in all patients, but there were no statistical difference, indicating that choosing age-stratified cutoffs did not improve the predictive characteristics in these study populations. However, compared with the same group of patients at the current age recommended cutoffs (125 ng/L for patients aged≤75 and 450 ng/L for those aged>75) [26], the accuracies of NT-proBNP for LV failure at the optimal cutoffs were markedly improved (all *p*<0.001). Table 3 shows the diagnostic characteristics of NT-proBNP for LV failure in patient with AECB patients.

**Table 3:** Diagnostic characteristics of NT-proBNP for LV failure in patients with AECB.

Cutoff (ng/L)	Sensitivity (%)	Specificity (%)	Accuracy (%)	PPV(%)	NPV(%)
All patients (n = 311)					
935.0*	94.4	68.2	74.3	47.2	97.6
Patients aged≤75 (n = 160)					
947.0*	93.9	78.0	81.3Δ	52.5	98.0
125.0**	100.0	25.2	40.6	25.8	100.0
Patients aged>75 (n = 151)					
2241.0*	76.9	80.4	79.5Δ	57.7	90.9
450.0**	100.0	35.7	49.4	35.1	100.0

* The present study optimal cutoff value;

** the current age recommended cutoff value.

Δ *p*<0.001, compared with the same group of patients at the current age recommended cutoff value for NT-proBNP.

NT-proBNP: N-terminal pro-B type natriuretic peptide; AECB: acute exacerbation of chronic bronchitis; LV: left ventricular; PPV: positive predictive value; NPV: negative predictive value.

Multivariate logistic regression analysis demonstrated that elevated NT-proBNP (≥935.0 ng/L) was a strong independent predictor of LV failure (odds ratio 28.37, 95% confidence interval 9.89 to 81.35, *p*<0.001). Another predictor was coronary heart disease. Table 4 shows the independent predictors of LV failure in multivariate logistic regression analysis

**Table 4:** Independent and mutually adjusted predictors of LV failure in multivariate logistic regression analysis.

Variable	Odds Ratio	95% confidence interval	*P* value
Elevated NT-proBNP (≥935 ng/L)	28.37	9.89 to 81.35	0.001
Coronary heart disease (yes or no)	3.13	1.03 to 9.51	0.0438

NT-proBNP: N-terminal pro-B type natriuretic peptide; LV: left ventricular.

### 4. NT-proBNP versus clinical judgment for diagnosis of LV failure

**Figure 3:**
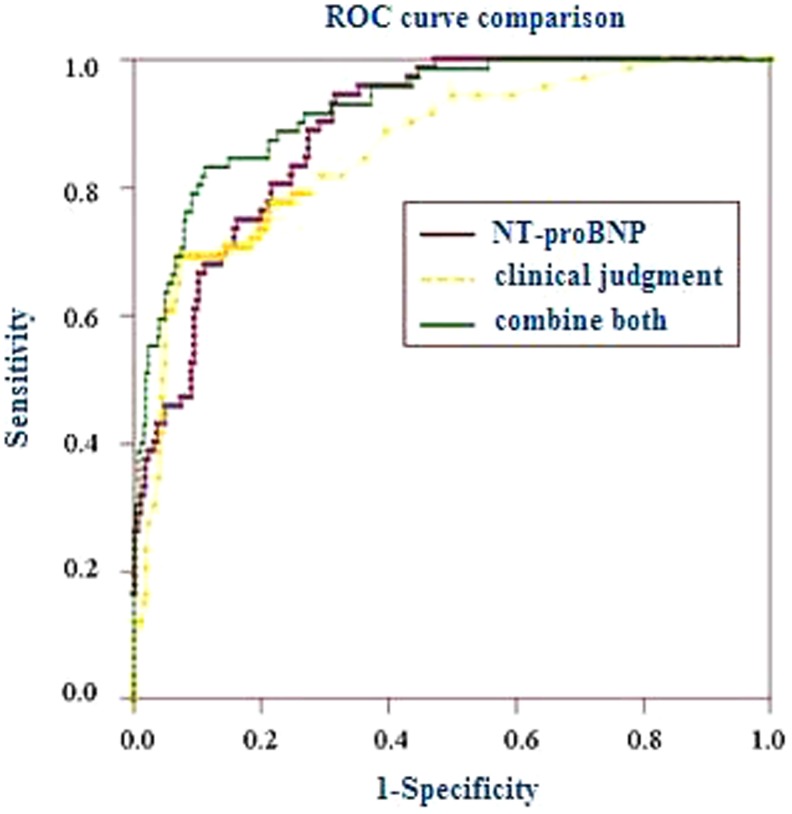
Comparison of receiver operating characteristic curves among NT-proBNP, clinical judgment and both combine for diagnosis of LV failure in patients with AECB. The area under the receiver operating characteristic curve (AUC) is 0.884 (95% confidence interval, 0.847 to 0.920) for NT-proBNP; 0.835 (95% confidence interval, 0.794 to 0.874) for clinical judgment alone; 0.923 (95% confidence interval 0.887 to 0.950) for both combine.

Table 5 shows the difference of basic variables between patients with and without LV failure. Based on these clinical variables, ED physicians used VAS to identify the probability of LV failure. Results showed, the high probability (≥80%) of LV failure was 41.7% (30/72) in patients with a final diagnosis of LV failure, and 5.4% (13/239) in those without the diagnosis. Conversely, the low probability (≤20%) of LV failure was 30.6% (22/72) in those with a final diagnosis of LV failure, and 72.8% (174/239) in those without. Using these clinical judgments alone, we performed ROC analyses and compared it with NT-proBNP test in identifying LV failure. The outcomes are shown in Fig. 3. NT-proBNP test was superior to clinical judgment alone (AUC 0.884 vs 0.835, *p* = 0.0294). Adding the results of NT-proBNP to those of clinical judgment alone for LV failure improved the global model fit, better discriminatory capacity with a significant increase in c-statistic value (AUC) and better calibration of the models with a higher P-value of Hosmer–Lemeshow test (Table 6), and also allowed a corrected reclassification in LV failure (Table 7).

**Table 5:** Basic variables for clinical judgment of LV failure.

	LV failure (n = 72)	No LV failure (n = 239)	*p*-value
Symptoms			
Nocturnal cough	54 (75.0%)	165(69.1%)	0.42
Fatigue	64 (88.9%)	209 (87.4%)	0.89
Orthopnea	51 (70.8%)	158(66.1%)	0.55
Functional class	3.1 (1.0–3.0)*	2.5 (1.0–3.0)*	0.04
Physical examination			
Peripheral edema	43 (59.7%)	58 (24.3%)	0.00
Elevated JVP	29 (40.3%)	24 (10.0%)	0.00
Hepatic congestion	10 (13.9%)	13(5.4%)	0.03
Enlarged heart	5(6.9%)	14 (5.8%)	0.95
Third heart sound	15 (20.8%)	3 (1.3%)	0.00
Wheezing	29 (40.3%)	120 (50.2%)	0.18
Rales	44 (61.1%)	67(28.0%)	0.00
Chest X-ray			
Cardiomegaly	48 (66.7%)	23 (9.6%)	0.00
Pulmonary edema	25 (34.7%)	8 (3.3%)	0.00
Hyperinflated lungs	9 (12.5%)	37 (15.5%)	0.60
Pleural effusion	20 (27.7%)	13 (5.4%)	0.00
Abnormality on ECG*	59.7%	30.1%	0.00

* Values are expressed as median (IQR).

**Table 6:** Discrimination and calibration of LV failure when NT-proBNP added to clinical judgment.

	clinical judgment alone	NT-proBNP combined with clinical judgment
c-statistic	0.835	0.927*
P (Hosmer-Lemeshow)	0.42	0.54

* *p*<0.001, compared with the clinical judgment.

NT-proBNP: N-terminal pro-B type natriuretic peptide; LV: left ventricular.

**Table 7:** Reclassification in LV failure when NT-proBNP added to clinical judgment.

	LV failure		Non-LV failure	
	True	False	True	False
clinical judgment alone (n)	35	22	217	37
NT-proBNP with clinical judgment (n)	41	8	231	31
Reclassified/categories*	6/35	14/22	14/217	6/37
Rate of correction (%)	17.1	63.6	6.5	16.2

* Categories: groups of predicted heart failure according to the clinical judgment alone.

NT-proBNP: N-terminal pro-B type natriuretic peptide; LV: left ventricular.

## Discussion

In the present study, we specifically examined the potential of plasma NT-proBNP to discriminate LV failure in AECB patients with no history of HF. Our results demonstrated NT-proBNP for LV failure in patients with AECB had a higher AUC, with a higher sensitivity and negative predictive value at an optimal cutoff value. The addition of NT-proBNP to clinical judgment would improve diagnostic accuracy for LV failure in patients with AECB. These data suggested that NT-proBNP still is a useful tool for identifying LV failure in patients with AECB.

HF is one of common complications in patients with AECB, and suspected as a cause of exacerbation in many such patients [6]–[8], [27], [28]. As there is the pre-existence of basic symptoms such as dyspnea and wheezing in patients with CB and these symptoms are relatively non-specific [29], which makes clinical judgment very difficult. Thus, the physician should carefully discriminate whether worsening dyspnea is the result of deterioration of pulmonary disease and/or the onset of HF. which is critically important as it will greatly influence therapeutic decisions. However, as demostrated in our study, emergency physicians were either uncertain or missed the diagnosis of LV failure in a substantial proportion of those ultimately proven to have LV failure, or those ultimately proven to have no LV failure.

Natriuretic peptides have been used world wide in diagnosis or exclusion of acute HF [9]–[12], and the utility of natriuretic peptides in congestive heart failure management has been stressed in the last European Society of Cardiology guidelines [30]. Thus, determining plasma NT-proBNP levels to identify LV failure may be appealing in patients with AECB. The analysis of this study is based upon an evaluation of the utility of plasma NT-proBNP measurement to offer a diagnostic service for patients with LV failure.

Our data demonstrated that NT-proBNP was a stronger predictor of LV failure in multivariate logistic regression analysis. Moreover, a diagnostic test with an AUC higher than 0.8 is commonly considered to be of clinical value. The greater AUC resulted from our study showed that NT-proBNP testing is useful for identifying LV failure in patients with AECB. In fact, our ROC analysis illustrated that using NT-proBNP test for identifying HF was more superior to clinical judgment alone, while adding the results of NT-proBNP to those of clinical judgment alone would significantly improve the diagnostic accuracy. These results were consistent with the previous (PRIDE) study by *Januzzi JL et al.* [31]. Importantly, the negative predictive values for LV failure were higher than 90.0% at the corresponding optimal cutoff values of NT-proBNP both in patients stratified by age and in all patients, suggesting that NT-proBNP is more useful as a “rule out” test in our study populations. As demonstrated by our study, addition of NT-proBNP to clinical judgment would help physicians to rule out more than half of the false LV failure patients. Similarly, *Gariani et al.* [21] assessed the diagnostic performance of BNP in detecting LV dysfunction in patients with no history of HF admitted for acute exacerbation of COPD. For a cutoff of 500 ng/L, they found a sensitivity of 62.0%, a specificity of 80.0%, a positive predictive value of 47.0% and a negative predictive value of 88.0%. In the present study, we assessed NT-proBNP instead of BNP and found NT-proBNP seemed to have more superior diagnostic performance to BNP. In addition, *Abroug et al.* [23] assessed the accuracy of NT-proBNP for the diagnosis of LV dysfunction in 148 selected patients with severe acute exacerbation of COPD. A cutoff of 1000 ng/L was accurate to rule out left-heart involvement with a sensitivity of 94%, a negative predictive value of 94%. Although these patients differed from our sample, and they tended to be 10 years younger on average as well as the symptoms of acute exacerbation presented more severe, the results of this study and ours are comparable.

Of note, the AUC of the NT-proBNP for detecting LV failure in our patients with AECB was somewhat lower than in previous studies of patients with suspected HF and patients with acute dyspnea attending the Emergency Department [31]–[34]. The possible reasons were as followings. Firstly, our patients had higher pulmonary pressure. Numerous studies have shown that in primary or secondary pulmonary hypertension, there is a supraphysiologic secretion of natriuretic peptides. Nagaya and coworkers showed that atrial natriuretic protein and BNP levels each correlated with mean pulmonary artery pressure, right atrial pressure, right ventricular end-diastolic pressure, and pulmonary resistance [35]. Secondly, there is the presenting feature of a wide range of other co-morbidities during exacerbation [29], while some of these co-morbidities, such as atrial fibrillation, renal dysfunction, may affect the natriuretic peptide levels [13], [19], [20]. Consistent with these findings, our study showed that pulmonary pressure, atrial fibrillation and renal function had independent effects on plasma NT-proBNP levels. Thirdly, during acute exacerbation, hyperinflation of the lung is associated with decreased cardiac function and lead to an increase of plasma BNP levels [36]. Because acute exacerbation is associated with a transient decrease in expiratory flow, which, in some cases, takes weeks to return to the baseline [37]. The radical decreases in expiratory flow may lead to increased air-trapping and hyperinflation of the lung [36]. In fact, our data showed that plasma NT-proBNP levels were elevated in patients with AECB, regardless of whether they have LV failure or not. This result was consistent with the recent subgroup analysis by *Inoue Y et al.* [38], who found that plasma BNP level during exacerbations was significantly higher than during stable disease. Fifthly, most of patients with newly detected LV failure were probably seen in an early stage of HF, which makes it plausible that the concentrations of NT-proBNP are lower than in patients with established HF [34], [39], because NT-proBNP production in the ventricles of the heart increases in response to increased intracardiac volume or pressure. Finally, although the distension of ventricular cardiomyocytes is generally considered the main mechanical stimulus, the production/secretion of BNP may be the result of a complex integration among mechanical, chemical, hemodynamic, humoral, ischemic, and inflammatory inputs [18]. Of these factors, some pro-inflammatory cytokines may be important pathophysiological mechanisms active in AECB patients. It is these differences that may influence the overall diagnostic accuracy, especially the specificity, of the NT-proBNP for detecting HF.

The limitation to the present study is this observational study was done at a single centre. We must acknowledge that the optimal cutoffs and the diagnostic characteristics of NT-proBNP assessed in our study are dependent on the patient population studied and the “gold standard” used for diagnosis of LV failure, and they also may be influenced by the assay method because the diagnostic information provided by NT-proBNP measurement might be method-dependent [40]. Another, the small sample size of our study limits the precision of our estimates. Therefore, one must be careful about generalizing the results to the entire population.

From our results, we concluded that, our results, along with other previous works, supported that NT-proBNP, readily available in most hospital settings, was of potential value for the identification of LV failure and confirmed a role of NT-proBNP measurement for ruling out LV failure in patients with AECB.
