# Correction: Utility of NT-proBNP for Identifying LV Failure in Patients with Acute Exacerbation of Chronic Bronchitis

**DOI:** 10.1371/annotation/b034b49e-ac17-4bd5-9ed8-42b51aca5e73

**Published:** 2013-04-22

**Authors:** Qing-ping Wang, Xiao-zhi Cao, Xue-dong Wang, Juan Gu, Li-min Wen, Li-ming Mao, Ping-nan Shan, Ai-guo Tang

There were errors in the order of author Affiliations. The correct list of affiliations is:

1 Department of Clinical Laboratory, The Shaoxing Hospital of China Medical University and Central Hospital of Shaoxing County, Shaoxing County, Zhejiang, PR China

2 Department of Clinical Laboratory, The Fifth People’s Hospital of Wuxi, Affiliated Hospital of Nanjing Medical University, Wuxi, Jiangsu, PR China

3 Department of Caroliology, Fuzhou General Hospital, Nanjing Command, PLA, Fuzhou, Fujian, PR China

4 Department of Clinical Laboratory, The Second XiangYa Hospital of Central South University, Changsha, Hunan, PR China 

